# Comparative analysis of right element mutant *lox *sites on recombination efficiency in embryonic stem cells

**DOI:** 10.1186/1472-6750-10-29

**Published:** 2010-03-31

**Authors:** Kimi Araki, Yuka Okada, Masatake Araki, Ken-ichi Yamamura

**Affiliations:** 1 Institute of Resource Development and Analysis, Kumamoto University, Honjo 2-2-1, Kumamoto 860-0811, Japan

## Abstract

**Background:**

Cre-mediated site-specific integrative recombination in mouse embryonic stem (ES) cells is a useful tool for genome engineering, allowing precise and repeated site-specific integration. To promote the integrative reaction, a left element/right element (LE/RE) mutant strategy using a pair of *lox *sites with mutations in the LE or RE of the *lox *sequence has previously been developed. Recombination between LE and RE mutant *lox *produces a wild-type *lox*P site as well as an LE+RE double mutant *lox *site, which has mutations in both sides and less affinity to Cre, resulting in stable integration. We previously demonstrated successful integrative recombination using *lox*71 (an LE mutant) and *lox*66 (an RE mutant) in ES cells. Recently, other LE/RE mutant *lox *sites showing higher recombination efficiency in *Escherichia coli *have been reported. However, their recombination efficiency in mammalian cells remains to be analyzed.

**Results:**

Using ES cells, we compared six RE mutant *lox *sites, focusing on their recombination efficiency with *lox*71. All of the RE mutant *lox *sites showed similar recombination efficiency. We then analyzed the stability of the recombined product, i.e., the LE+RE double mutant *lox *site, under continuous and strong Cre activity in ES cells. Two RE mutants, *lox*JTZ17 and *lox*KR3, produced more stable LE+RE double mutant *lox *than did the *lox*66/71 double mutant.

**Conclusion:**

The two mutant RE *lox *sites, *lox*JTZ17 and *lox*KR3, are more suitable than *lox*66 for Cre-mediated integration or inversion in ES cells.

## Background

**Figure 1:**
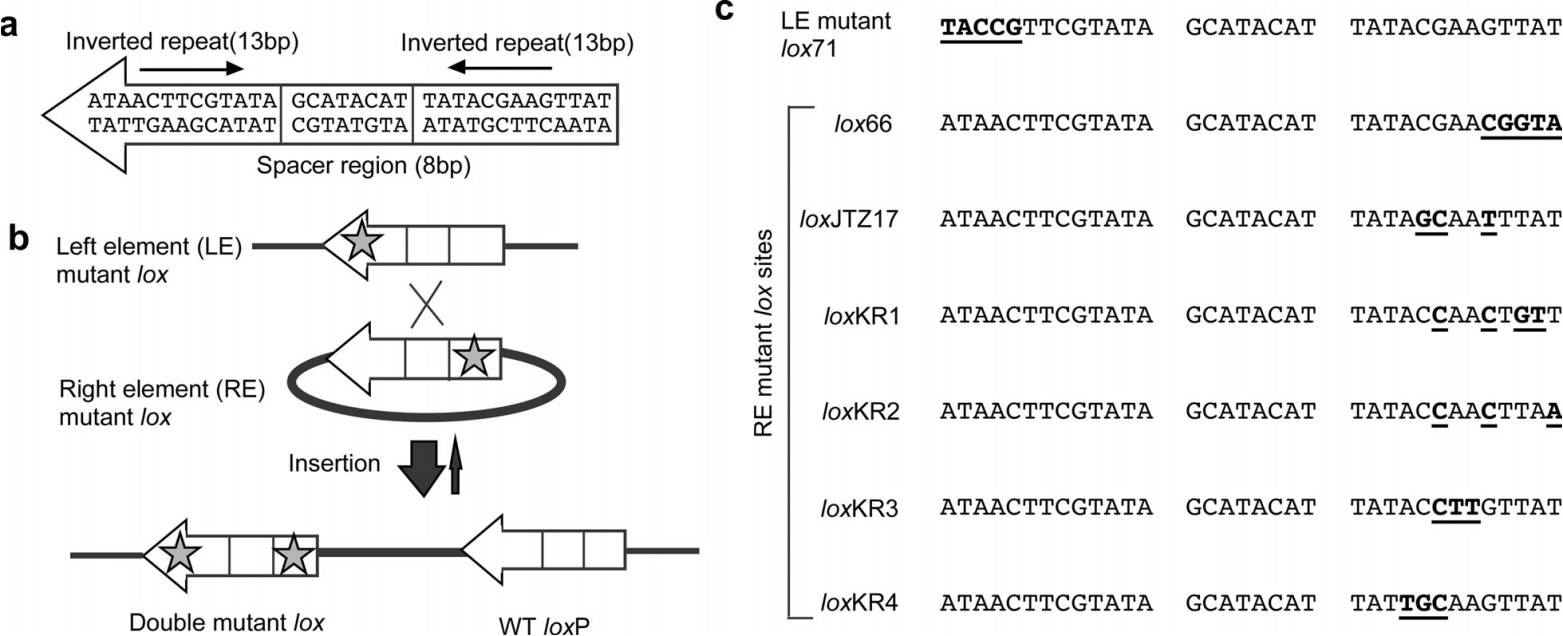
**LE/RE mutant *lox *system**. (a) Wild-type *lox*P sequence. The *lox*P site is composed of an asymmetric 8-bp spacer flanked by 13-bp inverted repeats that the Cre recombinase recognizes and binds to. (b) Integrative recombination with LE/RE mutant *lox *sites. The asterisks represent the mutations. RE mutants have mutations in the right inverted repeat region, and LE mutants have mutations in the left inverted repeat region. Through recombination between LE and RE mutant *lox *sites, a wild-type *lox*P site and a double mutant *lox *site carrying mutations in both ends are produced. The double mutant *lox *site has a lower affinity for the Cre enzyme; therefore, recombination of the product rarely occurs, and the recombination reaction tends toward integration. (c) Sequences of mutant *lox *sites used in this study. *Lox*71 carries 5 bp of mutation in the left element. The sequence of the right element of *lox*66 is complementary to the left element sequence of *lox*71. Mutated sequences are indicated by bold and are underlined.

The bacteriophage P1-derived Cre/*lox *recombination system has been extensively used to engineer the genome of experimental animals [1,2]. Cre recombinase recognizes a 34-bp element, termed *lox*P, which is composed of two 13-bp inverted repeats that serve as Cre binding sites, and an 8-bp spacer region that participates in strand exchange during recombination (Figure 1a) [3,4]. Depending on the relative orientation of the *lox *sites with respect to one another, the recombination reaction can result in excision, inversion, or integration.

Integrative recombination is useful for the production of transgenic animals or cells because any DNA of interest can be introduced into a chromosomally located *lox *site. However, integrative recombination between wild-type *lox*P sites is inefficient due to re-excision through intramolecular recombination [5]. Studies of mutated *lox*P sites have revealed that two classes of mutations can promote Cre-mediated insertion or replacement. One class consists of heterospecific *lox *sites carrying mutation(s) in the central 8-bp spacer region [6-8]. Recombination does not occur between two *lox *sites differing in the spacer region, whereas *lox *sites with identical spacer regions can be recombined efficiently. Recombination using heterospecific *lox *sites is termed "recombinase mediated cassette exchange (RMCE)" [9], in which one chromosomally preinserted DNA cassette flanked by two different heterospecific *lox *sites is exchanged for another cassette on a targeting plasmid flanked by the same kind of heterospecific *lox *sites. To date, *lox*511 [10], *lox*2272 [11], and *lox*5171 [12] have been successfully used in embryonic stem (ES) cells.

The other class is the left element/right element (LE/RE) mutant strategy using LE mutant *lox *carrying mutations in the left-inverted repeat region and RE mutant *lox *carrying mutations in the right-inverted repeat region [13]. Recombination between an LE mutant *lox *and an RE mutant *lox *results in the generation of a double mutant *lox *site having mutations in both ends and a wild-type *lox*P site. The double mutant *lox *site is not an effective substrate for Cre recombinase; therefore, the recombination reaction proceeds exclusively in one direction (Figure 1b). We have previously demonstrated successful integrative recombination using *lox*71 (an LE mutant) and *lox*66 (an RE mutant) in ES cells [14]. Moreover, two other groups have used *lox*71/66 to induce unidirectional Cre-mediated inversion [15,16].

Although the integrative recombination efficiency using *lox *71/66 is lower than RMCE efficiency using *lox*P and *lox*2272 [17], the advantage of the LE/RE mutant *lox *strategy is its simplicity. Only one LE or RE *lox *site is required as a target for integrative recombination. Recently, Thomson et al. performed mutational analysis of LE/RE mutant *lox *sites using *Escherichia coli *and identified two novel LE/RE mutant *lox*, *lox*JT15 and *lox*JTZ17, which showed approximately 1500-fold higher integration rates than *lox*71 and *lox*66 [18]. If these novel mutant *lox *sites could also improve integration efficiency in ES cells, they would be useful tools for Cre-mediated integration in mammalian genomes.

One application of the Cre/mutant *lox *integration system is gene trapping in ES cells. Our group, as well as two other groups (Database for Exchangeable Gene Trap Clones, Sanger Institute Gene Trap Resources and Bay Genomics), have constructed gene trap vectors incorporating a *lox*71 site and have generated over 20,000 gene trap cell lines, which are available to the academic community through the International Gene Trap Consortium database http://www.genetrap.org/index.html. With these trap clones, any DNA of interest can be inserted into a *lox*71 site and expressed under the control of the trapped gene promoter [19]. Therefore, in this study, we focused on screening for efficient RE mutant *lox *possessing better recombination efficiency with the *lox*71 site. Thomson et al. reported that the recombination efficiency between *lox*JTZ17 and *lox*71 was 10 times higher than that between *lox*66 and *lox*71. Although the 10-fold promoting effect is less than the 1500-fold effect obtained with *lox*JT15 and *lox*JTZ17, this improvement is considered sufficient because the recombination efficiency of *lox*66 and *lox*71 in ES cells is 2-16% [14]. Here, we used six RE mutant *lox *sites, including *lox*66, *lox*JTZ, and four newly synthesized RE mutant *lox *sites(*lox*KR1-4), and compared both their recombination efficiency and the stability of recombined products in ES cells.

## Results and Discussion

### Mutant RE lox sites

We synthesized four new RE *lox *sites (*lox*KR1-4) as well as *lox*JTZ17 (Figure 1c). *Lox*KR1 had four nucleotide mutations; of these, three mutated nucleotides were the same as the mutations in *lox*66, and one nucleotide was the same as the mutation in *lox*JTZ17. *Lox*KR2-4 were designed to have three mutations, as did *lox*JTZ17, but in different positions.

### Assessment of integrative recombination efficiency

**Figure 2:**
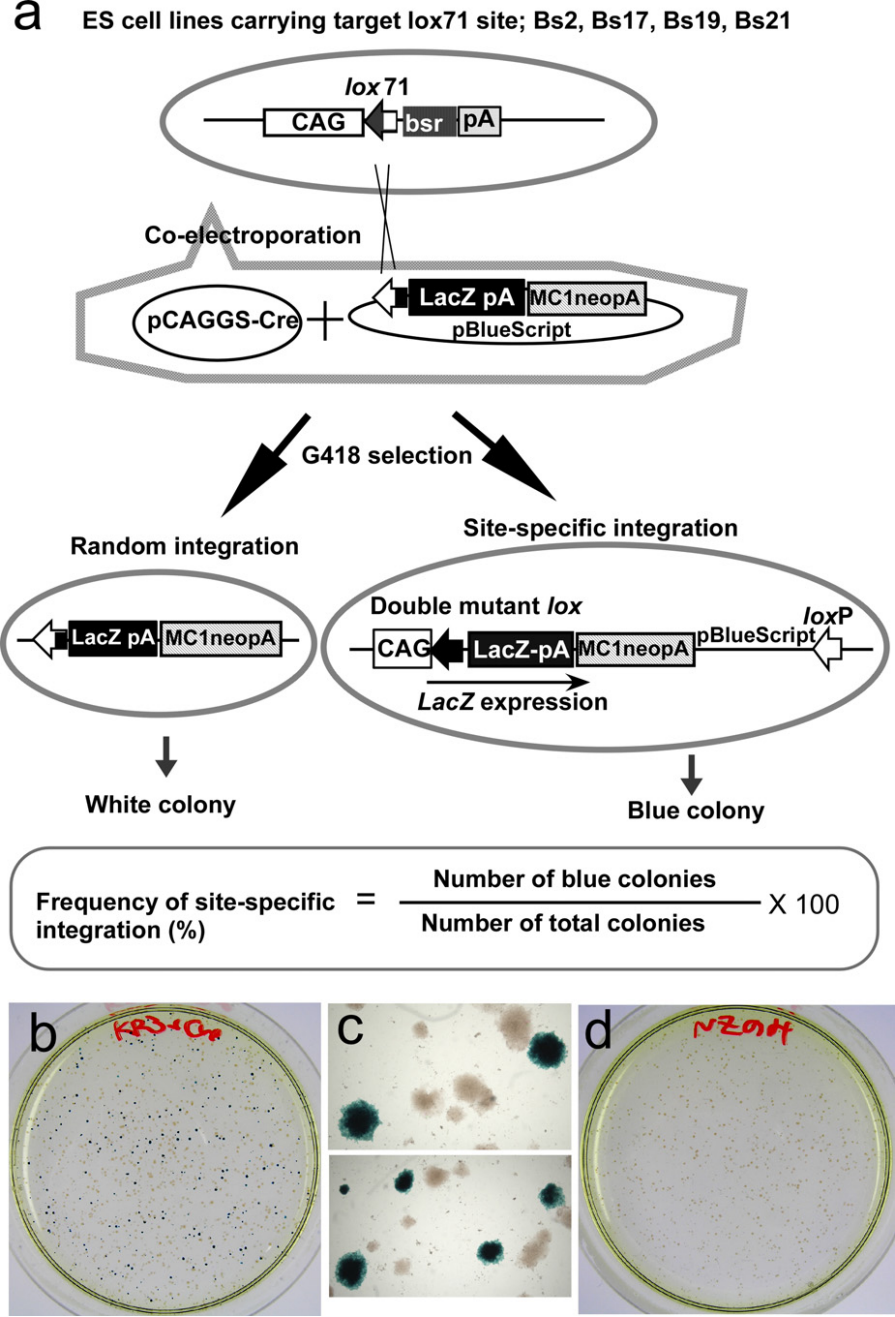
**Evaluation of recombination efficiency**. (a) Schematic experimental strategy. Four ES cell lines carrying a single copy of CAG promoter-*lox*71-bsr-pA were established and coelectroporated with an integration plasmid and the Cre expression vector, pCAGGS-Cre. After G418 selection, the colonies were stained with X-gal, and the percentage of blue colonies was scored as the site-specific integration efficiency. (b) Example of X-gal staining. Bs17 ES cells were coelectroporated with pCAGGS-Cre and pKR3NZneo, selected with G418 for 1 week, and then stained with X-gal. (c) Magnified photo of blue colonies. Most of blue colonies were stained uniformly. (d) X-gal stained plate electroporated without pCAGGS-Cre. Bs17 ES cells were electroporated with only pKR3NZneo. No blue colony appeared.

To test whether these RE *lox *sites could promote recombination efficiency in ES cells, we used the same strategy as described previously [14](Figure 2a). For the chromosomal target *lox*71 site, the CAG promoter-*lox*71-bsr-pA was introduced into ES cells. We isolated four ES clones—Bs1, Bs17, Bs19, and Bs21—that carried a single copy of the chromosomal target construct. Integration vectors comprised an RE *lox *site on the 5' side of the promoterless *NLSlacZ *gene and a selection marker gene, MC1-*neo*-pA. The ES clones were coelectroporated with the integration vector and the Cre-expression vector pCAGGS-Cre (in their circular forms) and then selected with G418. The *cre *gene was transiently expressed, mediating site-specific recombination between the chromosomal *lox*71 and the RE *lox *on the integration plasmid; this resulted in site-specific integration. The neo cassette is active regardless of the integration site; therefore, both random integrants and site-specific recombinants become drug resistant. However, in the present study, only the colonies where site-specific integration had occurred were stained blue with 5-bromo-4-chloro-3-indolyl β-D-galactopyranoside (X-gal) because the *NLSLacZ *gene was inserted downstream of the CAG promoter through Cre-mediated recombination. As shown in Figure 2b and 2c, most of the blue colonies were uniformly blue, indicating that recombination occurred in the early stage of colony formation. The percentage of blue colonies represented the frequency of site-specific integration. When only the integration vector was electroporated, no blue colonies appeared (Figure 2d).

### Site-specific integration frequencies

**Figure 3:**
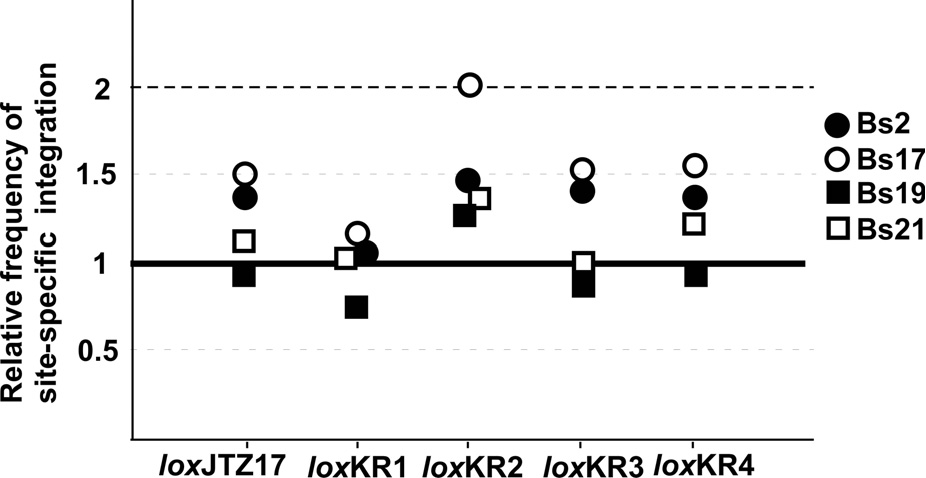
**Relative integration efficiency**. The efficiency obtained with the *lox*66 integration plasmid arbitrarily set at 1.

We constructed six integration vectors harboring different RE mutant *lox *and compared their recombination frequencies. Percentages of blue colonies, i.e., site-specific integration frequencies, in the four ES lines are shown in Table 1. The frequencies with the *lox*66-vector were 11-14%, and all other RE *lox *sites showed similar or slightly higher frequencies than were observed with *lox*66. Site-specific integration rates relative to the frequency with *lox*66 are shown in Figure 3. The differences observed were within a factor of 2, and there were no statistically significant differences among the RE *lox *sites (p = 0.37). Thus, the promoting effect of *lox*JTZ17 observed in *E. coli *was not clearly evident in ES cells. All new RE *lox *sites showed similar recombination efficiencies to *lox*JTZ17 and *lox*66.

**Table 1:** Frequency of site-specific integration

Cell line	*lox*66	*lox*JTZ17	*lox*KR1	*lox*KR2	*lox*KR3	*lox*KR4
Bs2	13.4 ± 4.3	18.0 ± 5.4	13.9 ± 2.5	19.2 ± 6.9	18.0 ± 2.4	17.5 ± 1.2
Bs17	11.3 ± 0.6	17.2 ± 2.2	13.3 ± 1.5	22.7 ± 3.3	17.3 ± 2.4	17.5 ± 3.7
Bs19	14.2 ± 2.9	13.3 ± 3.1	10.5 ± 1.8	18.0 ± 5.7	11.9 ± 3.4	12.9 ± 1.1
Bs21	13.8 ± 1.3	15.8 ± 2.6	14.4 ± 2.3	18.9 ± 3.6	13.9 ± 4.5	16.9 ± 2.7

Twenty micrograms of the integration plasmid and 10 μg of the Cre-expressing vector were coelectroporated. After drug selection for 7 days, colonies were stained with X-gal, and the percentage of positive colonies was scored as the frequency of site-specific integration. The means ± SD of three independent electroporations are represented.

### Assessment of stability of double mutant lox against Cre expression

Why did *lox*JTZ17 not increase the frequency of site-specific integration in ES cells? The major difference between the report by Thomson et al. [18] and the present study is the differing species of host cells: prokaryotic cells and eukaryotic cells, respectively. Prokaryotic cells are much smaller than eukaryotic cells. In addition, their genomic DNA is not separated by a nuclear membrane and has a more open structure than eukaryotic cells. Therefore, in prokaryotic cells, Cre proteins and *lox *sites should exist in much higher concentrations and meet and bind more frequently than in eukaryotic cells. The efficiency of integrative recombination in the LE/RE mutant *lox *system depends on the in-affinity of the LE+RE double mutant *lox *site to Cre protein. In this environment of prokaryotic cells, Cre proteins may be able to act on double mutant *lox *sites with incomplete levels of in-affinity for Cre protein. In our assay using ES cells and transient expression of the *cre *gene, the chance of a collision between Cre proteins and the *lox *site was much lower than in prokaryotic cells, and the double mutant *lox *site was exposed to Cre proteins for only a limited time. Therefore, Cre proteins may have disappeared before they could recombine *lox*P and LE+RE double mutant *lox*.

If the ineffectiveness of *lox*JTZ17 recombination in ES cells is due to the limited chance of a collision between Cre protein and *lox *sites, prolonged expression of Cre protein should affect the recognition and recombination of LE+RE double mutant *lox *sites. Therefore, we decided to examine the stability of LE+RE double mutant *lox *sites in ES cells under the continuous presence of Cre protein.

**Figure 4:**
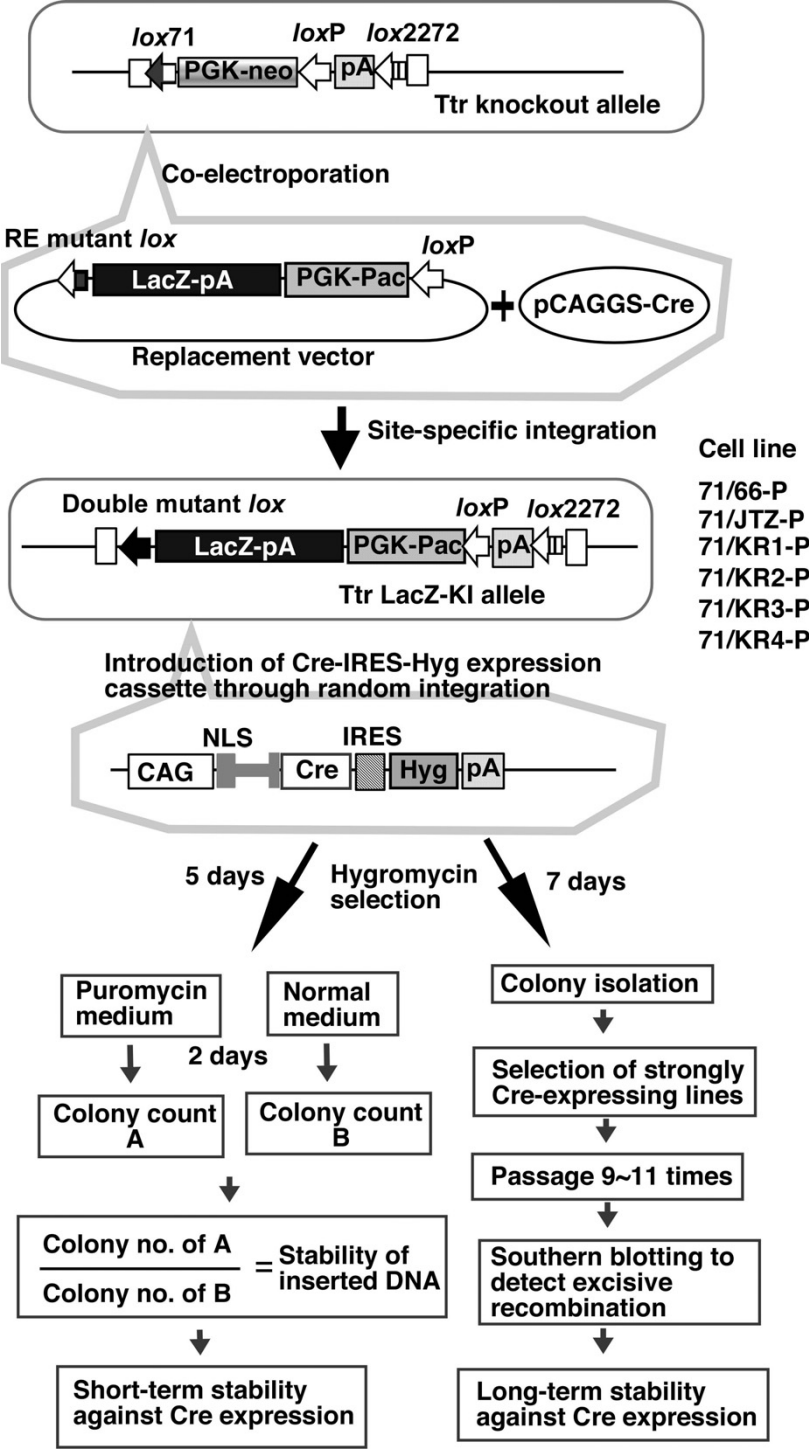
**Strategy to evaluate the stability of double mutant *lox***. In the Ttr-KO41 line, the *lox*71-Pgk-neo-*lox*P-pA-*lox*2272 cassette was inserted in the first exon of the mouse *Ttr *gene. Replacement vectors carried the RE mutant *lox*-LacZ-pA-Pgk-Pac-loxP. The Ttr-KO41 line was coelectroporated with the each replacement vector and the Cre-expressing plasmid; it was then selected with puromycin. Colonies were picked, and six sub-clones (71/66-P, 71/JTZ-P, 71/KR1-P, 71/KR2-P, 71/KR3-P, and 71/KR4-P) carrying different double mutant *lox *were established. To induce stable Cre-expression, the CAG-Cre-IRES-Hyg transgene was introduced into each sub-clone and selected with hygromycin. For the short-term stability assay, electroporated cells were divided into two plates, and after 5 days of hyg selection, one plate was selected with puro and the other with normal medium. The ratio of hyg^R ^puro^S ^colonies to hyg^R ^colonies represents the short-term stability of the double mutant *lox *site. For the long-term stability assay, Cre-expressing sub-lines with high levels were selected and passaged 9~11 times. Genomic DNA was prepared after 4, 7, 9, and 11 passages, and recombination at each stage was detected with Southern blotting.

The strategy used to evaluate the stability of the double mutant *lox *is shown in Figure 4. We used a Transthyretine (Ttr) knockout ES line (Ttr-KO41) in which the *lox*71-Pgk-neo-*lox*P-pA-*lox*2272 cassette was inserted into the first exon of the mouse *Ttr *gene. To produce LE+RE double mutant *lox*, six replacement vectors carrying the different RE mutant *lox*-LacZ-pA-Pgk-Pac-loxP were constructed, and the Ttr-KO41 line was coelectroporated with each replacement vector and a Cre-expressing plasmid. We expected that intramolecular recombination would initially occur, resulting in the removal of the Pgk-neo cassette from the genome and separation of the RE-mutant lox-LacZ-Pgk Pac cassette from the plasmid backbone. Cre would then mediate integrative recombination between chromosomal *lox*71 and the RE-mutant *lox *in the cassette. The *Pac *gene in the replacement vector does not have a pA signal; therefore, random integrants should be puromycin-sensitive, and cells should become drug-resistant only upon Cre-mediated targeted integration during which the *Pac *gene fuses to the pA signal. After coelectroporation, puromycin-resistant colonies were selected, and the integration pattern of the LacZ-pA-Pgk-Pac cassette was analyzed by polymerase chain reaction (PCR), Southern blotting, and sequencing (data not shown). Six sub-clones (71/66-P, 71/JTZ-P, 71/KR1-P, 71/KR2-P, 71/KR3-P, and 71/KR4-P) carrying different double mutant *lox *comprising the LE mutation of *lox*71 and the RE mutation of each RE mutant *lox *were established.

We first examined the short-term stability of these double mutant *lox*. Linearized CAG-Cre-IRES-Hyg cassette (pCAGNintCreIH) was introduced into each sub-clone, and Cre-expressing transformants were selected with hygromycin (hyg). Recombination between double mutant *lox *and *lox*P results in removal of the puromycin (puro) resistant genes; therefore, recombined cells become puro-sensitive. If recombination occurs soon after introduction of the CAG-Cre-IRES-Hyg cassette, hyg-resistant (hyg^R^) colonies should be puro-sensitive (puro^S^). In the formation of entire hyg^R ^puro^S ^colonies, recombination should occur before the first cell division, or, if formation occurs after the first cell division, recombination should occur in all of the daughter cells. Therefore, in this assay, it is possible to observe recombination within 24-48 h after exposure to Cre protein. To estimate the percentage of such hyg^R ^puro^S ^colonies, electroporated cells were divided into two plates. After 5 days of hyg selection, one plate was selected with puro and the other plate was fed with normal medium to obtain the hyg^R ^colony number. The ratio of hyg^R ^puro^S ^colonies to hyg^R ^colonies represents the short-term stability of double mutant *lox *site.

**Figure 5:**
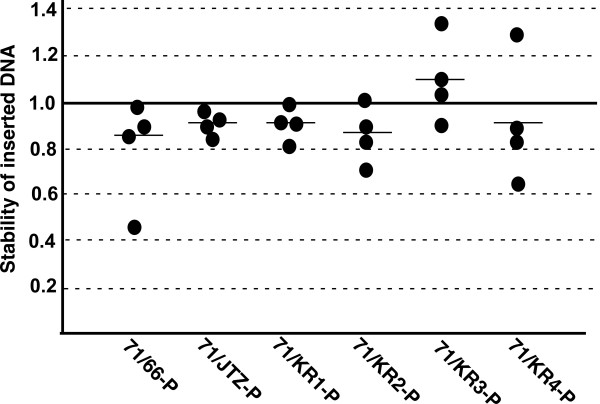
**Short-term stability of double mutant *lox *sites**. The ratios of hyg^R ^puro^S ^colonies to hyg^R ^colonies from four independent electroporations are represented. Bars indicate the means.

As shown in Figure 5, all double mutant *lox *showed over 85% stability of inserted DNA, indicating that recombination between double mutant *lox *and *lox*P occurs only slightly over a short time period (24-48 h) in ES cells. Although the/*ox*71/KR3 double mutant showed higher stability than did the others, there was no significant difference among double-mutant *lox *sites (p = 0.12). These results are consistent with the observation that there was no difference in integrative recombination.

**Figure 6:**
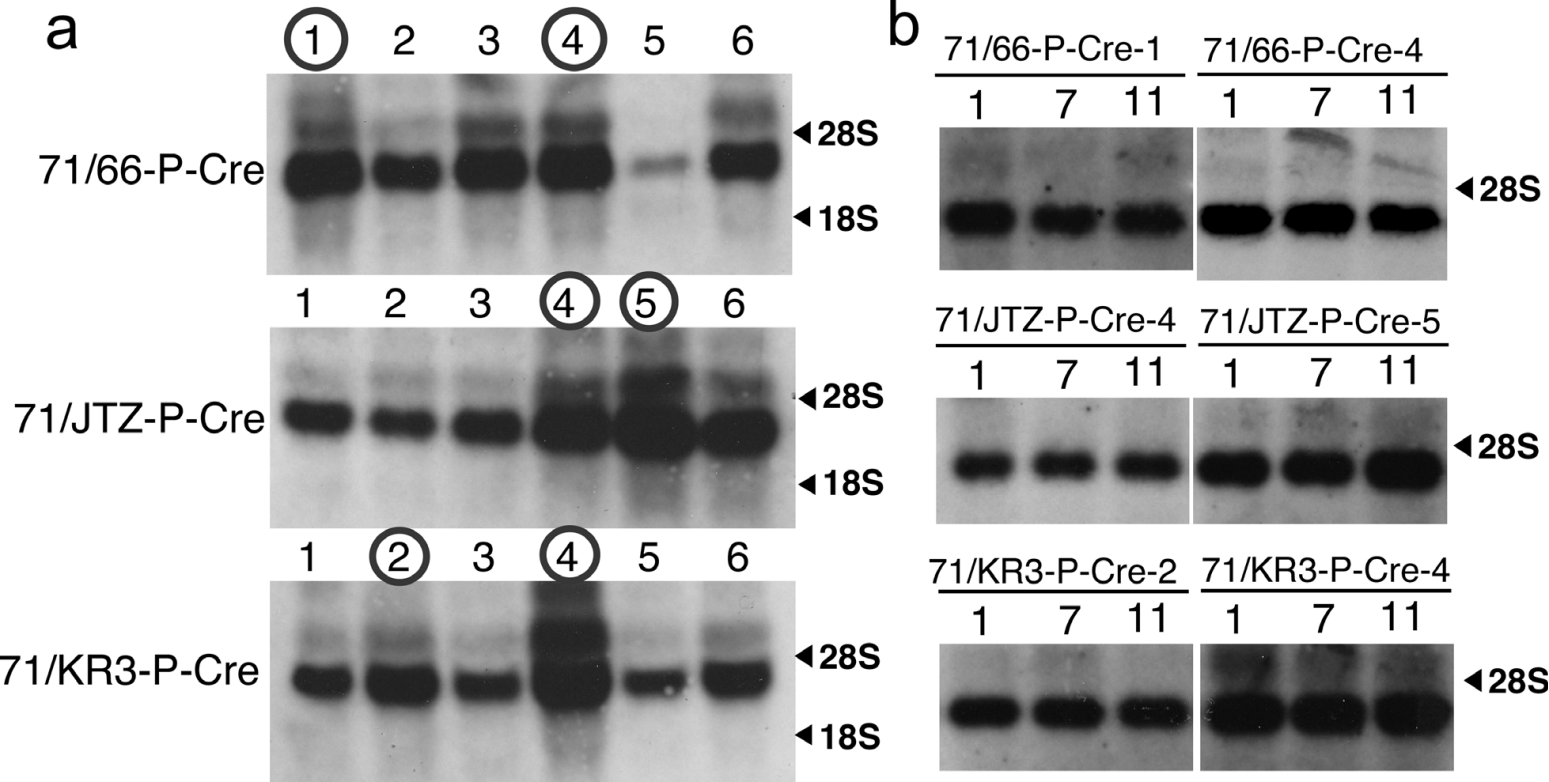
**Northern blot analysis of Cre-expressing sub-lines**. (a) Six sub-lines carrying the CAG-Cre-IRES-Hyg cassette were isolated from each of three double-mutant *lox *lines: 71/66-P, 71/JTZ-P, and 71/KR3-P. Ten micrograms of total RNA were subjected to electrophoresis and hybridization with a Cre probe. The two highest Cre-expressing lines selected from each of the six sub-lines are indicated by a circle. (b) Selected sub-lines were passaged 11 times, and RNAs from 1st, 7th, and 11th passage were prepared. Four micrograms of total RNAs were subjected to electrophoresis and hybridization with a Cre probe. The expression levels of Cre were maintained during passages.

**Figure 7:**
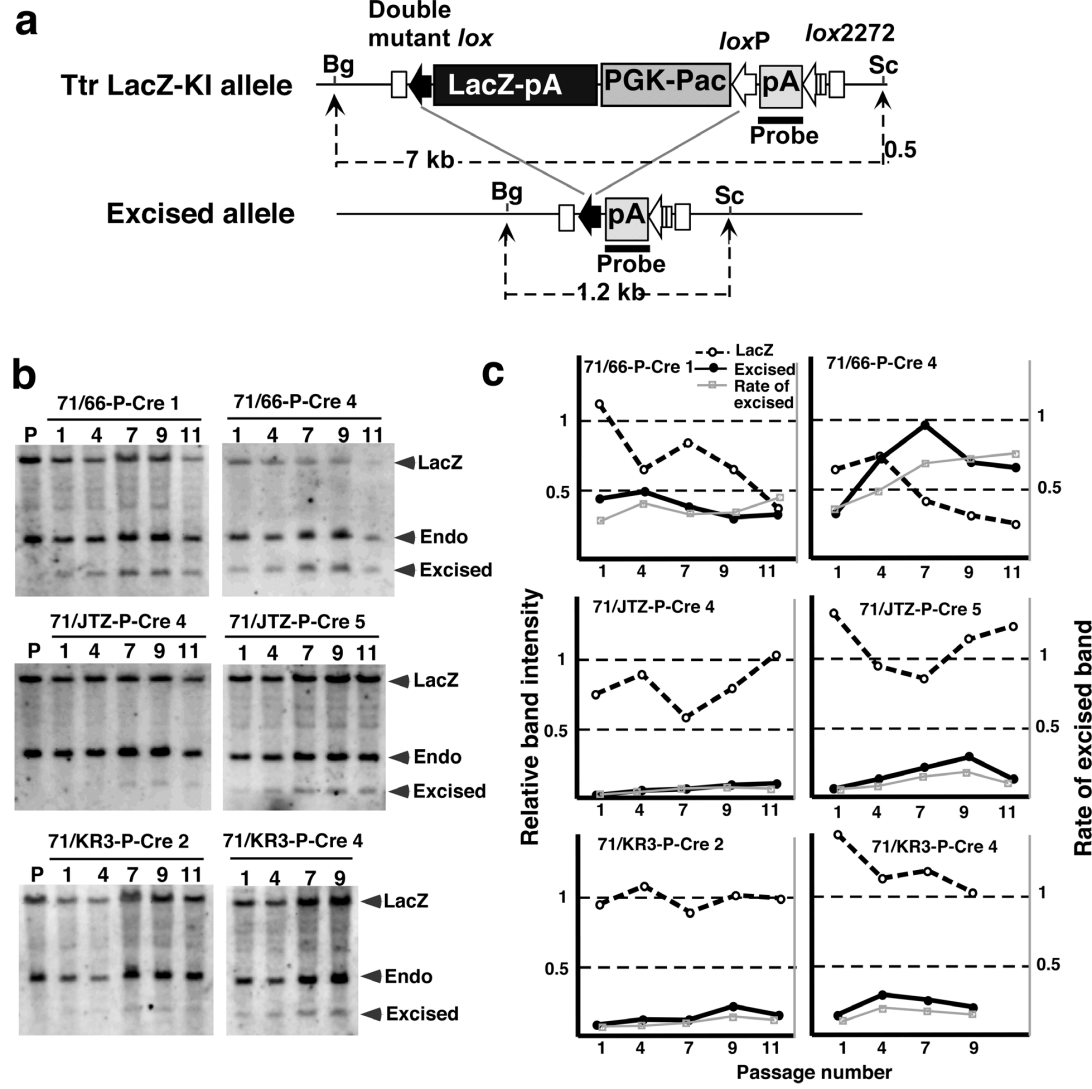
**Excising recombination between double mutant *lox *and wild-type *lox*P in strongly Cre-expressing sub-lines**. (a) Restriction endonuclease map of the targeted Ttr locus before (Ttr LacZ-KI allele) and after (Excised allele) Cre-mediated recombination. The pA signal of the mouse *Pgk *gene was used as a probe (indicated by the solid line). Fragment sizes are indicated. Bg, BglII; Sc, ScaI. (b) Southern blot analysis of Cre-expressing sub-lines. P represents the parental line before introducing the CAG-Cre-IRES-Hyg cassette. The numbers indicate passage numbers from the original cell stock. The positions of the bands from the nonexcised LacZ allele (LacZ), excised allele (Excised), and endogenous *Pgk *gene (Endo) are indicated by arrowheads. (c) Relative band intensities of the LacZ allele (hatched line) and excised allele (solid line) to the band intensities of the endogenous *Pgk *gene. The rate of excised band [ratio of excised band intensity to total (LacZ + excised) intensity] was calculated and is indicated by the gray line.

In this short-term assay, we could not detect whether recombination occurred later during colony formation in a limited population of the colony because such recombination generates countable, partly puro-resistant (puro^R^) colonies mixed with recombined puro^S ^cells and un-recombined puro^R ^cells. To establish the stability of double mutant *lox *sites against prolonged and strong Cre expression, we cloned and cultured Cre-expressing sub-lines, then examined the level of recombination by Southern blotting. Three double mutant *lox *lines, 71/66-P, 71/JTZ-P, and 71/KR3-P, were selected for the analysis of long-term stability (Figure 4, right side panel). After introduction of the CAG-Cre-IRES-Hyg cassette and colony formation, six Cre-expressing sub-lines from each parental line were isolated and stocked. Cre expression levels in the sub-lines were analyzed by northern blotting (Figure 6a), and the two highest Cre-expressing lines were selected from each double mutant *lox *parental line, as indicated in Figure 6a. The selected lines were then passaged 9~11 times from the original cell stock, and genomic DNA was prepared after 1, 4, 7, 9, and 11 passages. In order to confirm that the expression level of the *cre *gene was maintained during passage, total RNAs after 1, 7, and 11 passages were also prepared and subjected to northern blotting. As shown in Figure 6b, the *cre *gene was expressed with similar intensity during the 11 passages. Then, recombination between double mutant *lox *and *lox*P sites at each stage was analyzed by Southern blotting, as shown in Figure 7. In 71/66-P-Cre clones, the band for the excised allele was detected clearly at passage one, and the intensity became stronger with increasing passage number (Figure 7a). On the other hand, the bands for the excised allele in 71/JTZ-P-Cre and 71/KR3-P-Cre clones were faint (Figure 7b and 7c), indicating that *lox*71/JTZ and *lox*71/KR3 double mutant *lox *sites were more stable than *lox*71/66.

To estimate the rate of allele excision, band intensities relative to the band derived from the endogenous *Pgk *gene were measured (Figure 7d, solid and hatched lines), and the percentages of excised alleles were calculated (Figure 7d, gray line). In 71/66-P-Cre clones, the rates of allele excision were 48% (clone No. 1) and 74% (clone No. 4), suggesting that the *lox*71/66 double mutant is not highly resistant to re-recombination with *lox*P under continuous exposure to Cre expression. In 71/JTZ-P-Cre and 71/KR3-P-Cre clones, the rates of allele excision were under 21%, meaning that about 80% of double mutant *lox *sites were not recombined, even under the strong Cre expression forced by the CAG promoter (Figure 7d., middle and below).

**Figure 8:**
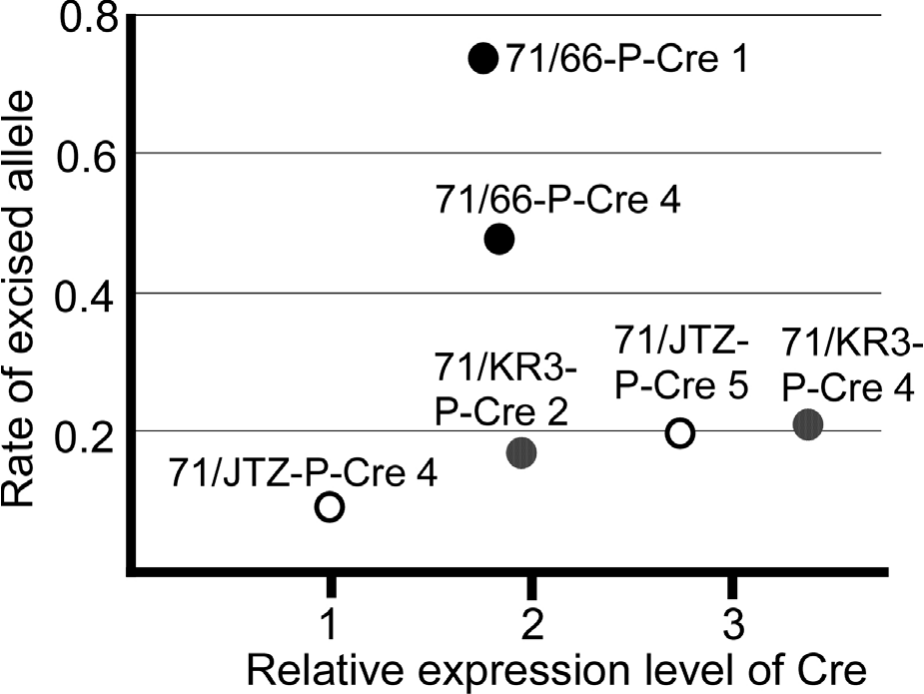
**Relation of excision and Cre expression level**. The northern blot X-ray film of Figure 6b was subjected to densitometry; the average of band intensities of samples from the 1st, 7th, and 11th passages were calculated; and the relative band intensity to 71/JTX-P-Cre 4, which showed the lowest expression, was calculated and plotted on the x-axis. The highest value of the excised allele rate calculated in Figure 7c was plotted on the y-axis. In 71/66-P-Cre clones, the recombination rate was apparently higher than in other clones.

In order to compare Cre expression levels in these Cre sub-clones, the band intensities shown in Figure 6b were measured by densitometry, and Cre expression level relative to 71/JTZ-P-Cre 4 was calculated. Figure 8 shows a scatter plot of the rate of excision (y-axis) and Cre expression level (x-axis) in each sub-clone. In 71/JTZ-P-Cre and 71/KR3-P-Cre clones, excision rate and Cre expression levels seemed to have a linear relationship with a similar correlation coefficient, suggesting that *lox*71/JTZ17 and *lox*71/KR3 have an in-affinity (stability) level similar to Cre protein. On the other hand, 71/66-P-Cre clones showed 2-4 times higher excision levels than did 71/JTZ-P-Cre and 71/KR3-P-Cre clones.

Thus, *lox*71/JTZ17 and *lox*71/KR3 are highly resistant to the Cre protein, but *lox*71/66 can be recombined with *lox*P when the *cre *gene is expressed strongly and constitutively. Therefore, if we used stable transformants of the *cre *gene for site-directed integration experiments, *lox*66 should show a lower efficiency because of its higher rate of re-excision.

## Conclusions

In this study, we screened for RE mutant *lox *sites showing higher recombination efficiency with *lox*71 using ES cells. Although we could not identify any RE mutant *lox *with a significantly higher efficiency than *lox*66, we found that two RE mutant *lox*, *lox*JTZ17 and *lox*KR3, produced more stable (less inactive) double mutant *lox *with *lox*71 than did *lox*66/71. These two mutant RE *lox *sites would therefore be more suitable than *lox*66 for Cre-mediated integration or inversion in ES cells.

## Methods

### Plasmids

Plasmids pCAGGS-Cre, pCAGlox71bsr, and plox66NZneo have been described previously [17,20]. The *lox*JTZ17 and *lox*KR1, 2, 3 and 4 sequences (Figure 1c) were synthesized, and the *lox*66 sequence of plox66neo was replaced by these synthesized *lox *sequences to produce pJTZNZneo, pKR1NZneo, pKR2NZneo, pKR3NZneo, and pKR4NZneo (RE *lox*NZneo plasmids). The p66NZPPacP plasmid was constructed by replacing the splice acceptor- enhanced green fluorescent protein (EGFP) cassette of p6SEFPPF [19] into the *LacZ *gene fused with the nuclear localization signal (NLS) derived from the SV40 large T gene (NLS-LacZ). The pJTZNZPPacP, pKR1NZPPacP, pKR2NZPPacP, pKR3NZPPacP, and pKR4NZPacP (RE *lox*NZPacP plasmids) were constructed by replacing the *lox*66 sequence with the *lox*JTZ17 and *lox*KR1, 2, 3, and 4 sequences, respectively. The sequences of all *lox *sites in these plasmids were confirmed by DNA sequencing.

The Cre-expression vector, pCAGNintCreIH, was assembled from components of pSP73 (Promega, USA), the CAG promoter [21], the NLS-splice donor (SD)-intron-splice acceptor (SA)-*cre *cassette [22], the internal ribosomal entry site (IRES) from the encephalomyocarditis virus (ECMV), the hygromycin-resistance gene, and the polyadenylation signal (pA) from the mouse *phosphoglycerate kinase-1 *(*Pgk*) gene.

### ES Cell cultures

ES cells were cultured in KSR-GMEM medium consisting of Glasgow Minimum Essential Medium (GMEM) (Sigma, USA) with 1× MEM nonessential amino acids (Gibco Invitrogen, USA), 0.1 mM β-mercaptoethanol, 1 mM sodium pyruvate, 1% fetal bovine serum (FBS; HyClone, Thermo Fisher Scientific Inc., USA), 14% Knockout™ Serum Replacement (KSR; Gibco Invitrogen), and 1100 U/ml leukemia inhibitory factor (LIF; ESGRO, Chemicon, USA). For neutralization of trypsin, FCS-GMEM in which the KSR in KSR-GMEM was replaced with FBS (final concentration, 15% FBS) was used.

ES cell lines (Bs2, Bs17, Bs19, and Bs21) carrying the target lox71 site were established from CGR8 [23] (Gift from Dr. Niwa) by introducing 10 μg of *Spe*I-digested pCAGlox71bsr plasmid DNA. ES cells (3 × 10^6 ^cells/0.8 ml in PBS) were electroporated using a Bio-Rad Gene Pulser (Bio-Rad, USA) set at 200 V and 960 μF and plated into two 10-cm plates. Blasticidin S selection was started after 48 h of electroporation at 4 μg/ml for 7 days, and colonies were picked, expanded, and stocked. Clones with a single copy integration were selected by Southern blotting analysis.

For Cre-mediated integration, ES cells were coelectroporated with 20 μg of RE *lox*NZneo plasmid and 10 μg of pCAGGS-Cre at 400 V and 250 μF. G418 selection at 600 μg/ml for 7 days was started after 24 h of electroporation. The colonies were then stained with X-gal.

The ES cell line Ttr-KO-41, which carries a *lox*71-Pgk promoter-neomycin phosphotransferase (neo) gene-*lox*P-pA cassette in the first exon of the Ttr gene (*Ttr*^*neo*^), has been described previously [24]. To obtain site-specific integrants of RE *lox*NZPacP cassette into the *Ttr*^*neo *^allele, Ttr-KO-41 ES cells were coelectroporated with 20 μg of RE *lox*NZPacP plasmid and 10 μg of pCAGGS-Cre at 400 V and 250 μF. Puromycin selection was started after 48 h at 2 μg/ml for 7 days. Colonies were picked and stocked. For electroporation of pCAGNintCreIH, ES cells were electroporated with 20 μg of XhoI-digested pCAGNintCreIH at 400 V and 250 μF and were fed 150 μg/ml of hygromycin B-containing medium after 24 h of electroporation. Hygromycin B selection was maintained for 5 days; we then changed to puromycin selection at 2 μg/ml or to normal medium for 2 days.

### Analyses of DNA and RNA

Cells were lysed with sodium dodecyl sulfate (SDS)/proteinase K, treated with 1:1 (vol/vol) phenol/chloroform, precipitated with ethanol, and dissolved in 10 mM Tris-HCl, pH 7.5/1 mM ethylenediaminetetraacetic acid (TE). Six micrograms of genomic DNA were digested with appropriate restriction enzymes, electrophoresed in a 0.9% agarose gel, and blotted onto a nylon membrane (Roche, Switzerland). Hybridization was performed using a DIG DNA Labeling Kit (Roche). The intensities of the obtained bands were determined using Printgraph AE-6920-MF (ATTO, Japan).

Total RNA was isolated from ES cells using Sepasol (Nakalai, Japan). Ten micrograms of total RNA were electrophoresed through 1.0% agarose-formaldehyde gels and transferred to a positively charged nylon membrane (Roche). Hybridization was performed using a DIG RNA Labeling and Detection Kit (Roche).

### Statistical analyses

The recombination efficiencies and relative number of blue or white colonies were evaluated by nonrepeated measures analysis of variance (ANOVA). Where a significant difference (p < 0.05) was identified, the differences were analyzed further with Student-Newman-Keuls (SNK) tests for multiple comparisons.

## Authors' contributions

KA contributed to the project conception and experimental design, carried out the experiments, and drafted the manuscript. YO participated in data production and analysis. MA participated in the experimental design and performed the statistical analysis. KY participated in coordination and helped with writing of the manuscript. All authors have read and approved the final manuscript.
